# Genetic and reproductive consequences of consanguineous marriage in Bangladesh

**DOI:** 10.1371/journal.pone.0241610

**Published:** 2020-11-30

**Authors:** Saeed Anwar, Jarin Taslem Mourosi, Yasir Arafat, Mohammad Jakir Hosen

**Affiliations:** Department of Genetic Engineering and Biotechnology, School of Life Sciences, Shahjalal University of Science and Technology, Sylhet, Bangladesh; University of Naples Federico II, ITALY

## Abstract

**Introduction:**

This study aimed to assess the prevalence, sociodemographic factors, reproductive consequences, and heritable disease burdens associated with consanguineous marriage (CM) in Bangladesh.

**Methods:**

A total of 7,312 families, including 3,694 CM-families, were recruited from 102 locales of 58 districts of Bangladesh. Using a standard questionnaire, we collected medical history and background sociodemographic data of these families. Family history was assessed by pedigree analysis. Fertility, mortality, secondary sex ratio, selection intensity, lethal equivalents were measured using standard methods.

**Results:**

The mean prevalence of CM in our studied population was 6.64%. Gross fertility was higher among CM families, as compared to the non-CM families (*p* < 0.05). The rate of under-5 child (U5) mortality was significantly higher among CM families (16.6%) in comparison with the non-CM families (5.8%) (*p* < 0.01). We observed a persuasive rise of abortion/miscarriage and U5 mortality rates with the increasing level of inbreeding. The value of lethal equivalents per gamete found elevated for autosomal inheritances as compared to sex-linked inheritance. CM was associated with the incidence of several single-gene and multifactorial diseases, and congenital malformations, including bronchial asthma, hearing defect, heart diseases, sickle cell anemia (*p* < 0.05). The general attitude and perception toward CM were rather indifferent, and very few people were concerned about its genetic burden.

**Conclusion:**

This study highlights the harmful consequences of CM on reproductive behavior and the incidence of hereditary conditions. It essences the need for genetic counseling from premarital to postnatal levels in Bangladesh.

## Introduction

Consanguineous marriage (CM) or cousin marriage is a type of interfamilial union, defined as the marriage between two blood-related individuals who are second cousins or closer (inbreeding coefficient ⩾ 0.0156) [1,2]. Over a billion of the global population live in communities where CM is a traditional and respected social trend of marital union [3,4]. The primary reasons for a preference for CM in communities with high consanguinity rates include maintaining the lineage solidarity of family, relative ease for the partners in finding a suitable spouse, backing the female status and improved relationships with in-laws, lowering the marital cost, enhancing the likelihood of getting better care for people in old age, and above all, better stability of marital relationship [1,2,5–9]. These factors seem to carry more significance in the context of protective and semi-protective cultures of many Middle Eastern, South and West Asian, and sub-Saharan African societies, which is why CM is most frequent in these countries [4,10]. Several reports have shown that the prevalence of CM in these areas ranges from some 7% to over 65%, predominantly between the first cousins (inbreeding coefficient ⩾ 0.0625) [2,10,11]. Although less frequent, CM is also seen in Europe, Australia, North America, and in many tribal populations [10,12].

Parental consanguinity is associated with an increased risk of autosomal recessive disorders and congenital anomalies in the offspring [10,13,14]. Besides, CM detrimentally affects the indicators of fetal survival and leads to the birth of progenies who are disadvantaged in terms of health parameters [13,15–19]. Parental consanguinity is a predisposing factor for many multifactorial complications, including obesity, cardiovascular disorders, diabetes, and some malignancies, which may influence the reproductive outcomes [11]. However, some reports refute the impact of CM on complex and multifactorial health issues [8,20]. The estimation of the overall adverse effects of CM is highly variable and perplexing to delineate from the epidemiological context [11,15,20].

Bangladesh is an overly populated (~170 million people) country with a lower-middle-income economy. With limited resources, the country is barely able to meet the living standards. The inadequacy of health education and awareness among a majority of the population makes it complicated for the country to ensure proper and equitable healthcare for its residents [21]. Usually, infectious and communicable diseases dominate the disease-related mortality in the country. However, in recent years, mortality due to chronic and non-communicable diseases has become a significant proportion of the disease burden in Bangladesh [22]. To note, Bangladesh is yet to start any genetic counseling services [23].

Over 90% of the population of Bangladesh are Muslims. It fosters a semi-conservative social structure with intense religious sentiments and societal bonding, which favors CM. However, little information is available on the prevalence, extent, and clinical implications of CM in the country. Only a few small-scale studies have attempted to determine the prevalence, socioeconomic, and birth-related effects of CM in some specific regions of the country [24,25]. We conducted a comprehensive survey to study the prevalence and obstetric, congenital, and clinical impacts of CM in Bangladesh. We also sought to investigate the CM-related perception, understanding of the risk information, and awareness among the Bangladeshi people.

## Materials and methods

### Ethical approval

This study was reviewed and approved by the Ethical and Animal Care Committee of the Shahjalal University of Science and Technology, Bangladesh. All the participants of this study were ethnically Bangladeshi. All participants provided informed consent according to the code of Ethics of the World Medical Association (Declaration of Helsinki).

### Population and study design

**Fig 1:**
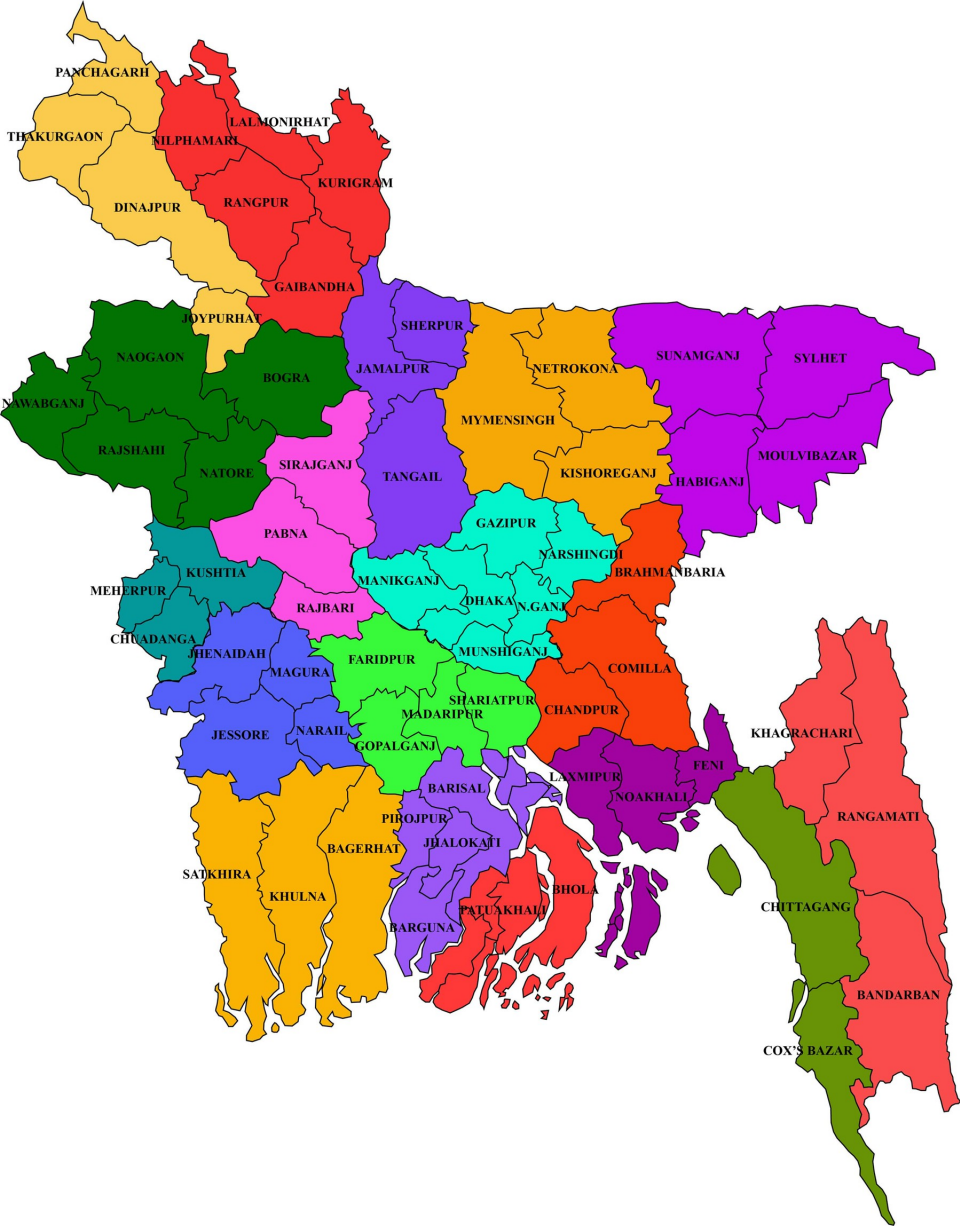
Relative geographical location of the study areas. Different colors refer to different zones. The base map depicted in this image is taken and modified from Schendel, 2009 [26].

We divided the whole country into 18 zones, including the hilly area in the southeastern part of the country (**Fig 1 and S1 Table**). The Chattogram hilly area (Khagrachari, Bandarban and Rangamati Hill Districts), which is residence to most of the tribal and first nation population of the country, we exclude them from our study. Due to a lack of communication, we could not survey in Lalmonirhat, Kurigram, and Meherpur. From each of the 17 zones (excluding Chattogram hilly area), we surveyed six systematically selected locales, with at least one locale from each district. Using a pre-tested, close-ended questionnaire, we conducted a house-to-house multiple indicator cluster survey (MICS) in the selected locales (**S2 Appendix and Fig 1**). All couples in the surveyed locales, who reported being married to their cousin (inbreeding coefficient of ≥ 0.0156) were enrolled in this study (test group). We administered the same questionnaire among the unrelated (non-consanguineous) couples of the same locales to constitute the control group (**S1 Appendix**). A team comprising of graduate students who majored in health and life sciences, clinicians, and local health officials conducted the questionnaire-based survey. From June 2017 to May 2019, we surveyed 102 locales of 58 districts of the country (**Fig 1**). To collect information about deceased individuals, we interviewed their spouse and first-blood relatives.

For each of the surveyed locales, we systematically documented all the phenotypic and clinical features, family history, and genealogical data. A registered medical doctor examined and helped extract the necessary data from the available medical documents and of the participants. In the case that medical documents of a proband were not available, and our accompanying doctor suggested laboratory testing for the diagnosis of the clinical condition, we collected specimens (blood and/or urine) from the proband(s) and their parents, and when needed/available, their unaffected siblings. The collected specimens were then tested using standard techniques at a registered diagnostic center or in our lab or one of our collaborators' lab following previously established protocols. Apart from the clinical information, we assessed the peoples' attitudes towards consanguinity, reproduction, awareness, and perception of risks. We also took data on the sociodemographic features, public health indices, and preferred type of medication (Allopathy/homeopathy/Ayurvedic). For a brief description of the case definitions, measures, and procedures, please see the **S1 Appendix**.

### Pedigree analysis

Genealogical information confirmed the CM pattern, which eventually helped to determine the mode of transmission for clinical phenotypes present in the children. We adopted Wright's path relationship method to calculate the coefficient of inbreeding for different mating types [27]. In non-consanguineous families (control), the coefficient of inbreeding was zero. Pedigrees or genograms were generated using Progeny, a free online tool (Progeny Genetics LLC, Delray Beach, FL; available at www.progenygenetics.com).

### Statistical analysis

Descriptive analyses were done to describe the general characteristics of the sample. Pearson's correlation coefficient determined correlations. Differences between groups were assessed using Mann–Whitney tests for non-normally distributed continuous ordinal data and Kruskal–Wallis tests for non-normally distributed variables with more than two groups. The mean ± SD between test (i.e., consanguineous) and control groups (i.e., non-consanguineous) were tested using the student's t-test. We used a logistic regression analysis (odds ratio, 95% confidence interval) to compare child mortality rates between different categories. Analysis of variance (ANOVA) and χ2-test revealed significant differences. Statistical significance was checked at 95%, 99% and 99.9% confidence levels (*p* < 0.05, *p* < 0.01 and *p* < 0.001, respectively). All data were analyzed in SPSS version 18 for Windows 10.

## Results

### Prevalence of consanguineous unions

During the two-year-long survey program, we surveyed 102 locales of 58 districts of the country where a total of 55,646 couple lives, including 3,694 consanguineous couples. The aggregate of the population in our studied locales were 292,994, of which, 18,269 were representatives of the consanguineous families. The unrelated couples enrolled in this study as controls included a total of 3,618 non-consanguineous families consisting of 17,411 individuals (**Table 1**).

**Table 1:** General features of the studied population.

Features	Numbers (n)
Total population	292,994
Total number of couples living in the surveyed locales	55,646
Consanguineous couples living in the surveyed locales	3,694
Non-consanguineous couples living in the surveyed locales	51.952
Control couples enrolled in this study	3618

The rates of CM ranged from 2.33% to 41.35% in the surveyed locales, with a mean rate of 6.638% (**Tables 2** and **S2**). The highest incidence rate (41.35% of 208 couples; 62.79% of consanguineous marriages between first-cousins) was observed in a village in Gafargaon, Mymensingh, while the lowest incidence rate (2.33% of 344 couples; 50% of consanguineous marriages between first cousins) was recorded in a village in Terokhada, Khulna. Division-wise, the lowest prevalence of consanguinity was recorded in Khulna (3.31%), while we observed the highest prevalence in Sylhet (8.76%) (**S2 Table**). Marriage between two first cousins was the most frequent type (66.19%) of CM. Among the first cousin unions, we observed no note-worthy preference for either parallel or cross-cousin marriages; however, we recorded a general predilection for matrilateral marriages (*p* < 0.01) (**Table 2)**. Besides, data showed a higher prevalence of CM in the rural areas compared to the urban areas (*p* < 0.01) (**S2 Table**). Our study revealed that the mean inbreeding coefficient in the progeny of the present generation was 0.0492 (**S2 Table**). From a religious point of view, CM seemed almost entirely a Muslim-dominated customary in Bangladesh, as all our interviewed couples were Muslims, except only one Hindu consanguineous couple in Moulvibazar.

**Table 2:** Prevalence of different types of marriages.

Relationship	Number (n)	Percentage (%)
All types of marital unions	55,646	100
Non-consanguineous	51,952	93.362
Consanguineous	3,694	6.638
Double first cousins	16	0.433
First cousins	2445	66.188
Parallel matrilateral	604	24.703
Cross matrilateral	673	27.526
Parallel patrilateral	621	25.399
Cross patrilateral	547	22.372
Other degree cousins ^a^	1233	33.378

^a^ Spouses not first cousins but have some blood relation with an inbreeding coefficient of greater than or equal to 0.015625

**Fig 2:**
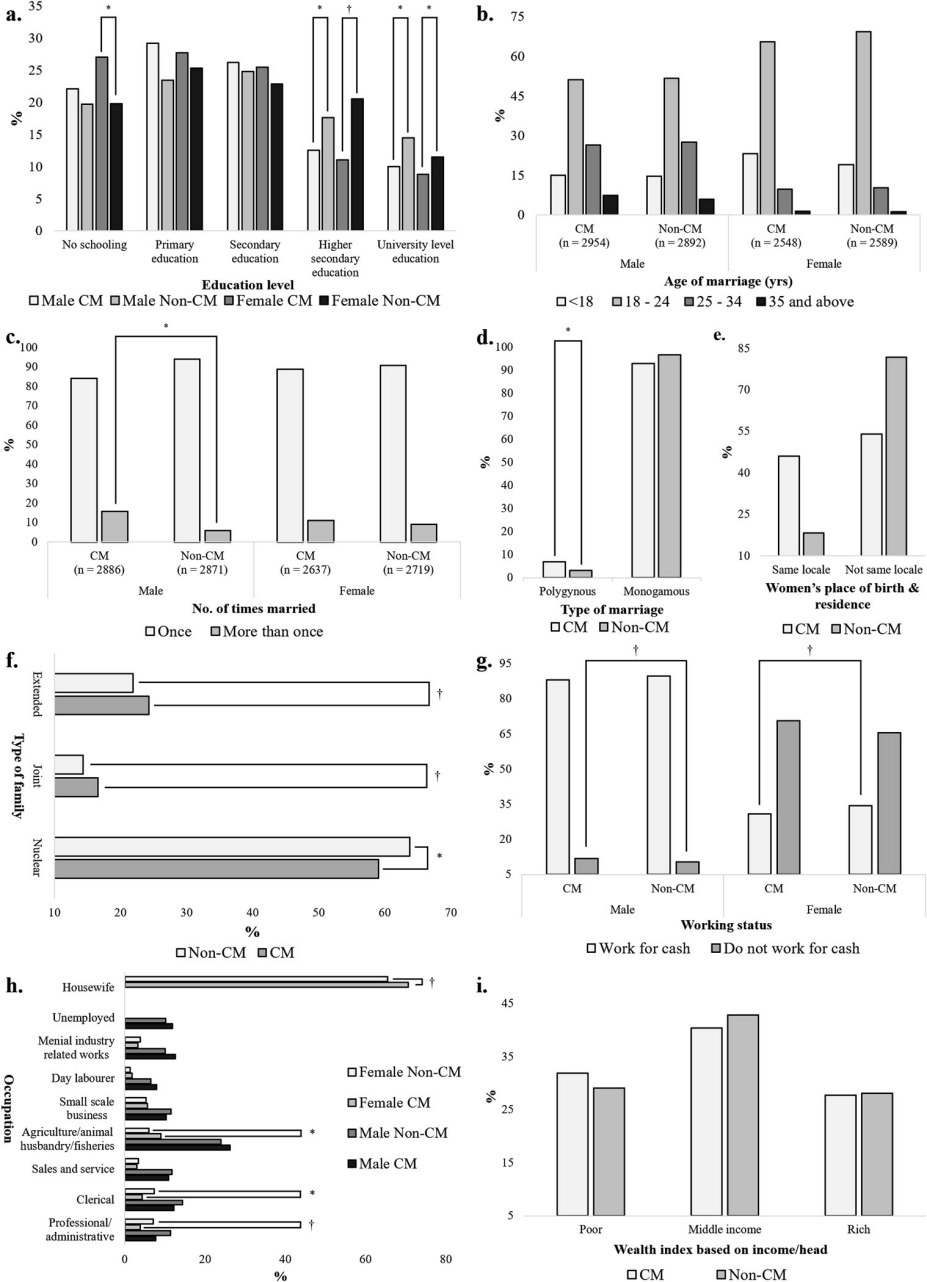
Background sociodemographic status of CM and non-CM participants. a) Education levels of CM and non-CM individuals. For both males and females, we have seen higher participation in post-secondary and university level education by the non-CM individuals. b) Age distribution at marriage. No notable difference between CM and non-CM individuals were seen. c) The number of times individuals got married. The occurrence of more than once marriage was more prevalent in CM males. d) Type of marriage practiced. We observed a higher occurrence of polygynous marriage in the CM group. e) Women's place of birth and residence. CM females more tend to live in the same locale as the locale they were born. f) Type of families. More non-CM couples preferred to live in nuclear families. g) Working status. The number of females working for cash income was significantly different among CM and non-CM groups. h) Occupational distribution of CM and non-CM couples. For both male and female, non-CM individuals were more involved with professional, administrative, and clerical occupations. i) Wealth index based on the per head yearly income of families. 41.61% (40.42% in CM and 42.81% in non-CM) of all families represented the middle-income category. There was no significant difference between the wealth index of CM and non-CM families. * *p* < 0.05; ^†^ *p* < 0.01.

Analysis of literacy and socioeconomic status revealed a significant difference between education levels in CM and non-CM groups (**Fig 2**). Whether male or female, nearly 1/4^th^ of the consanguineously related individuals received no schooling, and higher education was significantly more frequent in the non-CM individuals (**Fig 2A**). Although the mean age at marriage was not significantly different between CM (24.0 and 21.2 years in male and female respectively) and non-CM (23.9 years in male and 21.3 years in female) individuals, there was a substantial variation across age cohorts of CM and non-CM couples (**Fig 2B**). It appears that the youngest cohort of females of <18 years of age was significantly more prominent in CM than their non-CM counterparts (*p* < 0.01). Besides, we observed that more CM males were present in the oldest cohort of >35 years of age (*p* = 0.003). We tried to find an association between the age of individuals and CM; however, a χ^2^ test revealed no significant relation. No significant difference in the overall socioeconomic status of the CM and the non-CM group was evident (**Fig 2I**). However, our study revealed that there were fewer CM people, both male and female, involved in any work to earn cash (*p* < 0.05) (**Fig 2G**). Occupation-wise, we found that fewer CM females were involved in professional or administrative and clerical responsibilities, which usually require higher institutional education (*p* < 0.01) (**Fig 2H**). In contrast, more CM individuals, both male and female, were involved with agricultural, animal husbandry, and fisheries-related occupations (*p* = 0.022 and *p* < 0.01 respectively).

CM couples were more apt to live in the joint (stem/sibling) or extended families. In contrast, non-CM couples showed a higher preference for forming nuclear families (**Fig 2F**). Surprisingly, we found that CM is less likely to be stable than its non-CM counterparts (**Fig 2**). Our analysis indicated that there were higher proportions of polygynous and more than once marriage incidence among the consanguineous couples than among their non-consanguineous counterparts (**Fig 2C** and **2D**). However, women born and residing in the same locale after marriage have higher rates of CM than women having different places of birth and residence after marriage (**Fig 2E**).

### Influence of CM and sociodemographic factors on reproductive behavior

**Fig 3:**
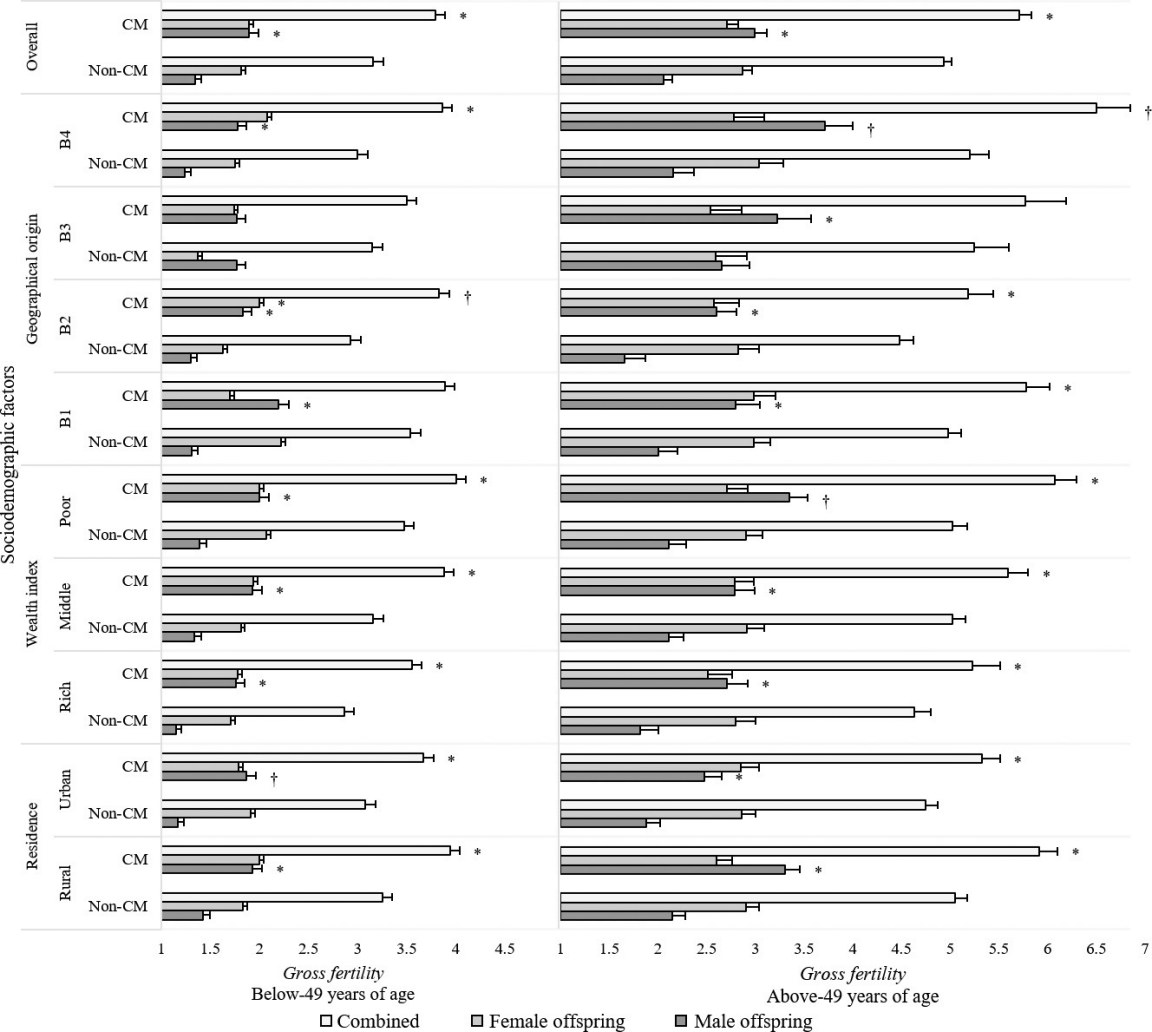
Influence of CM, sex of offspring, age of mother, and sociodemographic factors on gross fertility. B1, B2, B3, and B4 denote Northern Bengal, Eastern Bengal, Central Bengal and Southern Bengal geographical regions. * Significantly different from non-CM at *p* < 0.05, using a two-tailed students t-test. ^†^ Significantly different from non-CM at *p* < 0.01, using a two-tailed students t-test.

Our analysis revealed higher gross fertility for male offspring among the CM groups as compared to non-CM groups (**Fig 3**). We observed an overall higher gross fertility in the above-49 mothers as compared to the mothers below-49. Notably, a significantly lower gross fertility rate among both below-49 (3.16 ± 0.124) and above-49 (4.931 ± 0.178) non-CM mothers (*p* < 0.05) was found (**Fig 3**). The relationship between reproductive behavior and other sociodemographic factors, including residence (rural vs. urban), wealth index, geographical origin (between divisions), was also assessed; however, no statistically significant association was observed.

### Influence of CM and sociodemographic factors on children mortality under the age of 5

**Fig 4:**
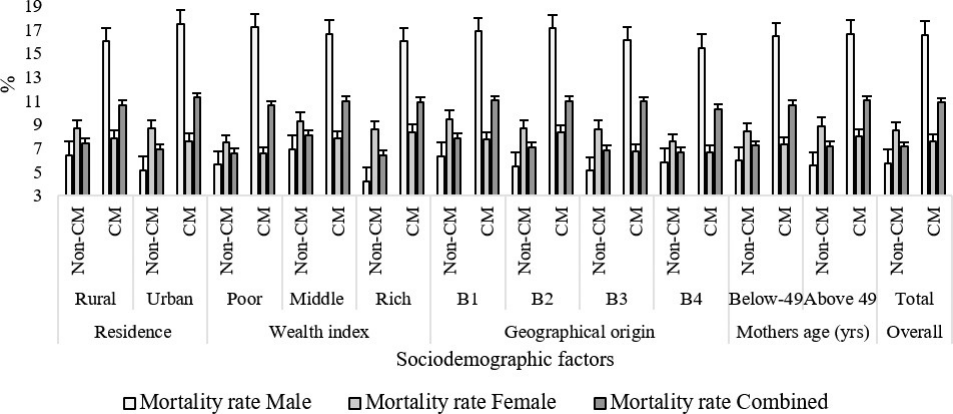
Impacts of CM and sociodemographic factors on U5 child mortality. CM families exhibit a higher rate of U5 mortality as compared to their non-CM counterparts. B1, B2, B3, and B4 denote Northern Bengal, Eastern Bengal, Central Bengal, and Southern Bengal geographical regions. * Analysis of variance (ANOVA) revealed a significant difference between CM and non-CM at *p* < 0.05; ^†^ ANOVA revealed a significant difference between CM and non-CM at *p* < 0.01.

Analysis of an aggregate of 32,117 birth cases, including 17,480 from CM families, revealed that the rate of child mortality under the age of 5 years (U5 mortality) was remarkably higher among CM families (16.62%, n = 2905) as compared to non-CM groups (5.77% n = 844) for all different categories (*p* < 0.05) (**Fig 4**). However, considering the sociodemographic factors, e.g., area of residence, wealth index, geographical origin, and mothers age, the rate of U5 mortality was significantly higher in the CM groups (~16.6%) than their non-CM counterparts (~5.8%) (*p* < 0.01) Notably, the U5 mortality rate in male children was very high in CM families coming from all different backgrounds (*p* < 0.01). Our analysis indicates that there is a negligible effect of different sociodemographic factors on U5 mortality; however, consanguinity has an irrefutable impact.

### Influence of CM on secondary sex ratio

**Fig 5:**
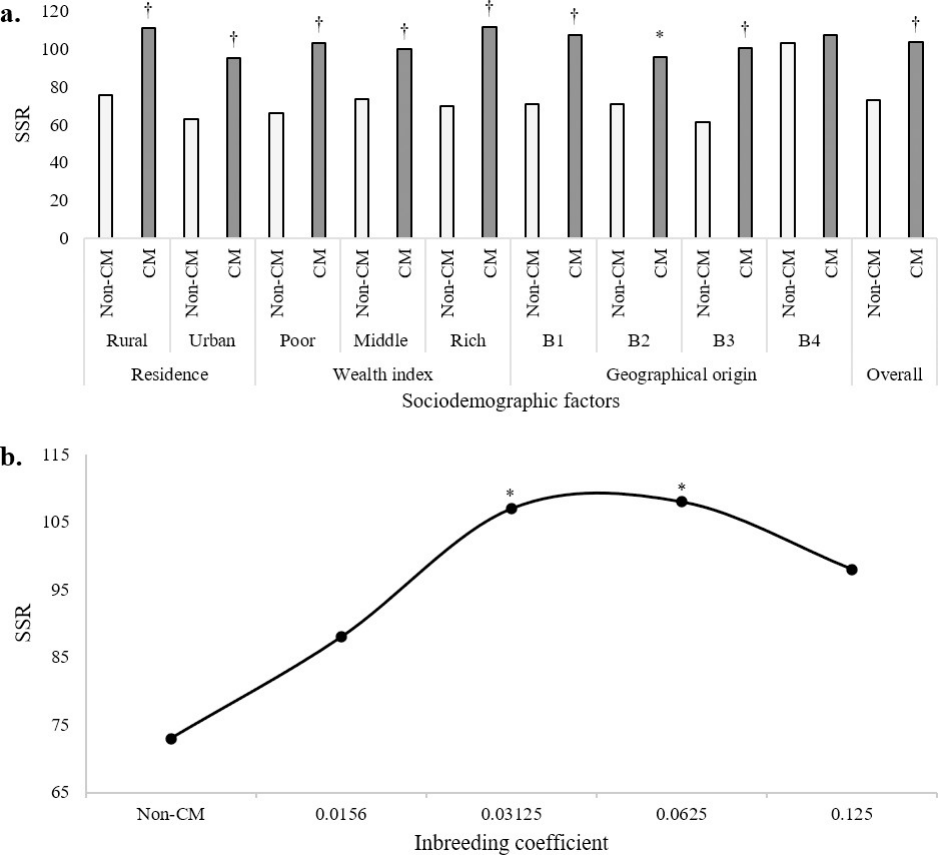
Impacts of CM on the Secondary Sex Ratio (SSR). a) A significantly increased SSR (number of males/100 females at birth) among CM families was seen, as compared to the non-CM group for residence, wealth index, and geographical origins. B1, B2, B3, and B4 denote Northern Bengal, Eastern Bengal, Central Bengal, and Southern Bengal geographical regions. b) To a certain level (F = 0.0625), SSR rose with an increase in the coefficient of inbreeding. Further increase in the inbreeding coefficient caused a decline in SSR value. * Significantly different from non-CM at *p* < 0.05; ^†^ Significantly different from non-CM at *p* < 0.01.

The number of males per 100 females at births (secondary sex ratio, SSR) was significantly increased among CM families as compared to the non-CM group for different sociodemographic factors (*p* < 0.01) (**Fig 5A**). Except for the families originated from the Southern part of the country, SSR was higher among CM families across all different factors we analyzed. Also, SSR rose significantly to a certain level with the increase in homozygosity (F = 0.0625), then declined with a further increase in inbreeding coefficient (F > 0.0625) (**Fig 5B**). Overall, the outcomes of this study reflect a considerable effect of consanguinity on SSR.

### Effect of CM on selection intensities

**Fig 6:**
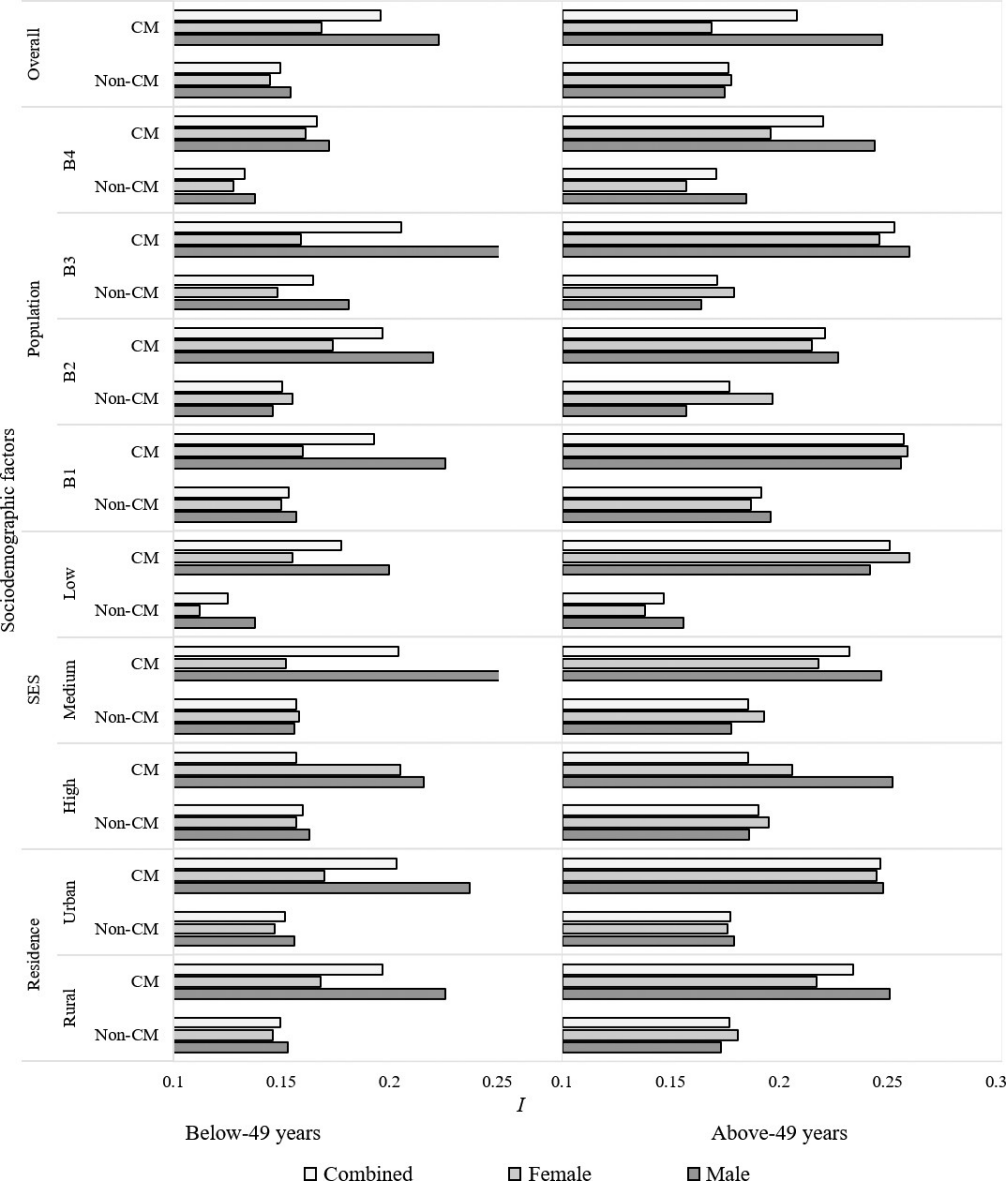
Differences in selection intensity between CM and non-CM families. *I* value was higher in the CM groups as compared to the non-CM groups, especially in the males. B1, B2, B3, and B4 denote Northern Bengal, Eastern Bengal, Central Bengal, and Southern Bengal geographical regions.

A lower value of selection intensity (*I*) was observed for both the CM and non-CM women (**Fig 6**). However, while comparing against the non-CM groups, the offspring from CM showed higher values of selection intensity (*I*) across all the residential and geographical backgrounds and wealth index. Besides, our analysis obtained higher values of I for male offspring (*I* = 0.222) than their female counterparts (*I* = 0.169). The results were all similar for both the mothers below-49 years of age and above-49 years of age; however, values were relatively higher in the above-49 years of age group (0.196 compared to 0.208).

### The odds ratio for miscarriage/abortion and U5 mortality rates increases with degrees of consanguinity

The miscarriage/abortion and U5 mortality rates showed higher odds ratios (OR) with the increasing degrees of consanguinity (**Table 3).** For both miscarriage/abortion and U5 mortality, all first and double first cousin categories had a significant rise in odds ratios (*p* < 0.05). Besides, U5 mortality has significantly increased odds ratio in the first cousin once removed group (*p* < 0.01). These outcomes suggest that the potential risk for child mortality, both in prenatal and postnatal stages, increases with homozygosity.

**Table 3:** Logistic regression analysis contrasting miscarriage/abortion and U5 mortality rates with different categories of CM. The potential risk for miscarriage/abortion and U5 mortality rate rises with the increasing degree of consanguinity.

Inbreeding coefficient	Miscarriage/Abortion			U5 Mortality		
	OR*	95% Cl	*p* value	OR*	95% Cl	*p* value
0.0156	1.1363	0.9012 to 1.4329	0.28	1.1314	0.9322 to 1.3732	0.2114
0.03125	1.2664	0.9541 to 1.6810	0.1021	1.2366	1.0622 to 1.4396	0.0062
0.0625	1.4328	1.0816 to 1.8981	0.0122	1.3428	1.0522 to 1.7136	0.0178
0.125	1.5768	1.3213 to 1.8818	< 0.01	1.4468	1.1511 to 1.8185	0.0015

OR: Odds ratio; Cl: Confidence interval (lower bound, upper bound)

* Calculated using non-CM as a control group

### Child mortality increases with the levels of homozygosity

**Fig 7:**
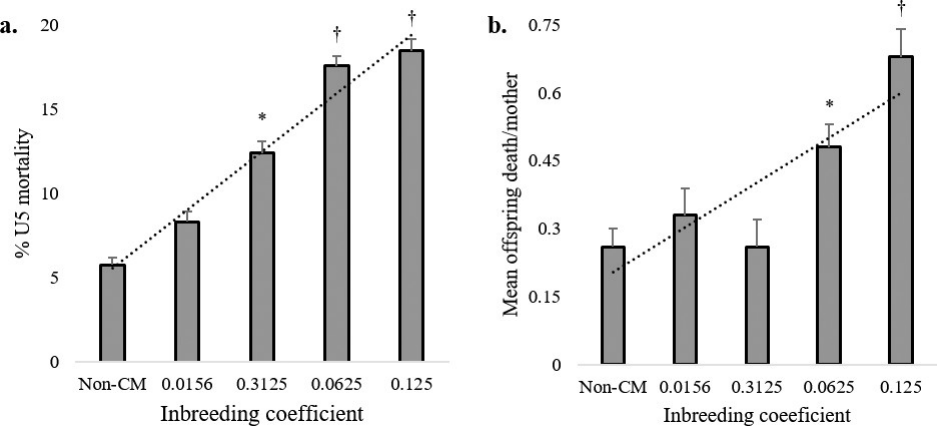
U5 mortality with increasing degrees of inbreeding. The (**a**) U5 mortality rate; (**b**) Mean child mortality per mother with the coefficient of inbreeding. Both (a) and (b) shows inflations in the U5 mortality rate and the mean number of child deaths per mother with increasing levels of homozygosity. * Analysis of variance (ANOVA) revealed a significant difference between CM and non-CM at *p* < 0.05; ^†^ ANOVA revealed a significant difference between CM and non-CM at *p* < 0.01.

Considering the inbreeding coefficient as a measure of the level of homozygosity, the U5 mortality rates increased almost proportionally with higher levels of homozygosity (**Fig 7**). To the increasing inbreeding coefficient, we observed a linear increment rate of the U5 mortality rate in the CM group (**Fig 7A**). This study found the highest rate of U5 mortality in the offspring of double first cousins (inbreeding coefficient = 0.125), while the least was observed for the non-CM group (inbreeding coefficient < 0.0156) (*p* < 0.01). The mean of offspring death per mother raised remarkably with the increased levels of homozygosity. The child mortality per mother rose markedly with increasing inbreeding coefficients (**Fig 7B**). We observed the highest mean death of offspring per mother in the double first cousin group (inbreeding coefficient = 0.125), while the least mean was reported in the non-CM group (inbreeding coefficient < 0.0156) (*p* < 0.01). These results depict that the harmful impacts of consanguinity increase with the degree of inbreeding and levels of homozygosity.

### Autosomal and X-linked genetic loads rise with increasing degrees of consanguinity

In all categories of CM, we observed higher values of lethal equivalents per gametes (LEG) for autosomal inheritance than X-linked inheritance (**Table 4**). Also, the genetic load increased proportionally with the degree of consanguinity. In our analysis, first cousins had markedly inflated load of consanguinity (LOC), while the second cousins showed the lowest value.

**Table 4:** Lethal equivalents per gametes and estimates of genetic load due to consanguinity.

Type of CM	Autosomal		X-Linked		LOC
	F	LEG	F	LEG	
Double first cousin	0.125	0.1292	0.1710	0.0944	0.797
First cousin	0.0625	0.2235	0.1203	0.1161	1.0538
First cousin once removed	0.03125	0.1411	0.0780	0.0565	0.9128
Second cousins	0.0156	0.0420	0.0478	0.0137	0.5745

F = inbreeding coefficient

### Parental consanguinity is associated with the higher frequency of genetic diseases in children

**Table 5** shows the pattern of genetic diseases in children of CM and non-CM couples. Pedigree analysis revealed that 903 (6.2%) of the 14,575 live children (24.6 ± 13.0 years, range: 01–63 years) of the consanguineous couples had health complications, which might have been predisposed in them genetically. The effect was significant as compared to their non-consanguineous counterparts (n = 280, 2.03% of 13793 live children; 26.5 ± 12.9 years, range: 01–59 years) (*p* < 0.05). The most predominant genetic complication was represented by congenital malformations, which accounted for 178 cases. Sickle cell disease and thalassemias were the most frequent examples of single-gene diseases. Besides, higher rates of several multifactorial diseases in the CM-group were observed, which includes bronchial asthma, epilepsy, hearing defect, kidney disorder, mental disorders, and vision-related complications. A total of 84 children, including 57 from the CM group, showed comorbid conditions.

**Table 5:** Parental consanguinity and genetic diseases in children from all participants. Offspring of CM couples had significantly higher proportions of genetic diseases as compared to their non-CM counterparts.

Disease/Symptoms	CM	Non-CM	OR	95% CI	*p-*value*
Bronchial asthma	130	39	3.17	2.22, 4.54	< 0.01
Diabetes	28	15	1.77	0.94, 3.31	0.0713
Disability	178	53	3.18	2.34, 4.32	< 0.01
Epilepsy	35	11	3.01	1.53, 5.93	0.0008
Hearing defect	93	34	2.59	1.75, 3.83	< 0.01
Heart disease	55	14	3.72	2.07, 6.68	<0.01
Kidney disease	47	12	3.71	1.97, 6.98	0.0501
Lactose intolerance	29	12	2.29	1.17, 4.48	0.0131
Mental disorder	28	9	2.94	1.39, 6.24	0.0031
Mental retardation	32	10	3.03	1.49, 6.16	0.0013
Sickle cell anemia	81	24	3.19	2.03, 5.03	< 0.01
Thalassemias	32	11	2.75	1.39, 5.46	0.0025
Thyroidism	65	20	3.08	1.86, 5.07	< 0.01
Tumor	46	16	2.72	1.54, 4.80	< 0.01
Undifferentiated sex	1	0	-	-	-
Vision problem	80	27	2.80	1.81, 4.33	< 0.01
Comorbid conditions	57	27	2.00	1.26, 3.16	0.0025
Total children affected with genetic diseases	903	280	3.05	2.67, 3.48	< 0.01

OR: Odds ratio; Cl: Confidence interval (lower bound, upper bound)

*Calculated from χ² tests

### Perturbed congenital malformations and parental consanguinity

Among the congenital malformation, congenital heart diseases (45.51%, n = 81) and physical anomalies (38.2%, n = 68) were found most prevalent and significantly associated with consanguinity (p < 0.01) (**Table 6**). The congenital physical anomalies included clinodactyly (07), ectrodactyly (04), syndactyly (04), achondroplasia (05), preauricular pits (09), cleft lips (14), imperforate anus (04), shortness of the fourth metacarpal (08) or metatarsal bones (06), atresia (05), and sacral dimples (04). Two of the CM children had a comorbid condition with preauricular pits and clinodactyly. We also found two other comorbidity cases where one CM and one non-CM child had cleft lips and sacral dimples. Among the other congenital malformations, nine hydrocephalus and eight neural tube defect cases observed; though, there was no significant difference between CM and non-CM groups (**Table 6**).

**Table 6:** Pattern of congenital anomalies associated with parental consanguinity.

Type of malformation	CM	Non-CM	*p*-value*
Congenital heart disease	81	22	< 0.01
Hip dysplasia	20	8	0.0337
Hydrocephalus	5	4	0.8019
Neural tube defects	4	4	0.9378
Physical anomalies	68	15	< 0.01
Total children affected with congenital malformations	178	53	< 0.01

*Calculated from χ² tests

### No significant difference in public health condition between CM and non-CM families

**Fig 8:**
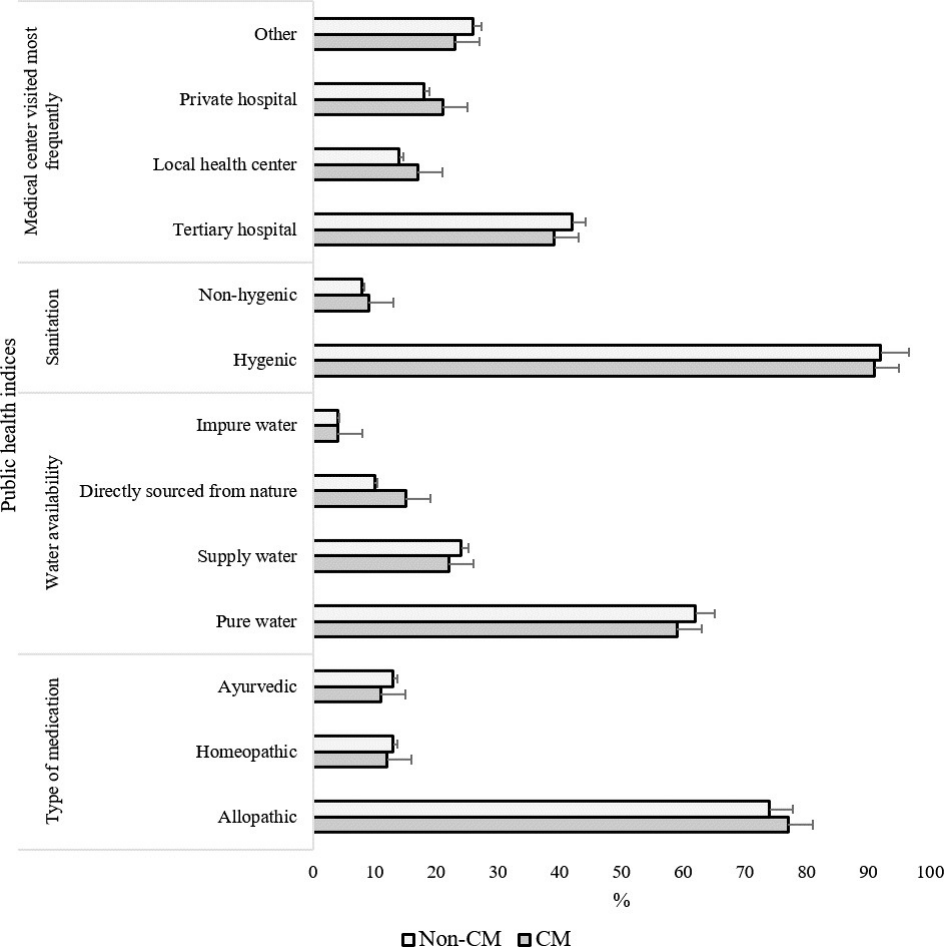
Comparison of public health indices between CM and non-CM families. At a 95% confidence level, no significant difference was observed between CM and non-CM groups.

Analysis of public health parameters revealed no significant difference between the CN and non-CM families (**Fig 8**). This analysis suggests that the deleterious outcomes reported in the CM families were not due to poor public health indices.

### Attitudes towards CM

**Fig 9:**
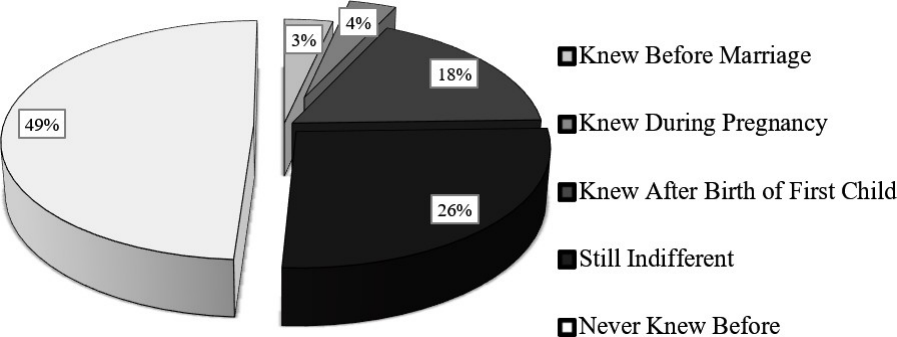
Level of knowledge and attitude towards consanguineous marriage among the CM couples. Almost half of the respondents had no idea about the detrimental effects of CM. 26% of the couples who knew about the impact of consanguinity before or learned from our team during the interviews were still indifferent.

On the detrimental effects of consanguinity, an indifferent state of mind among the CM couples was observed (**Fig 9**). Almost half of the respondents (49%) had never received any information on the genetic consequences of CM. In comparison, only 7% had at least some knowledge about the potential complications before getting exposed to a casualty of hereditary issues. When we provided them information about the genetic consequences of CM, over one-fourth of our studied couples (26%) were apathetic. Besides, our study revealed that almost 75% (n = 2770) of the total marriage decisions (CM) were taken from within the family (i.e., arranged marriage), and the rest were based on the choice of individuals and/or love affairs. In comparison, the percentage of arranged marriage was significantly lower in the non-CM families (n = 2203, 60.88%) (*p* < 0.01).

## Discussions

CM is a mode of marital union where the spouses share the genetics of a reasonably recent ancestor [28]. The offspring of CM may experience an increased risk of genetic disorders, congenital anomalies, and early childhood mortality [6,15]. However, it is still one of the preferred modes of marital union in many communities, including Bangladesh [29]. This mode of marital union is not uncommon in some African, Israeli, and tribal communities, though most prevalent throughout the Muslim-dominant Middle Eastern, West and South Asian countries [12]. Indeed, throughout all Islamic recorded history, it has been a norm and remains so until today [30]. Many reports have discussed the significance of the customary; some consider it an essential feature of the Middle Eastern kinship system, while others perceive CM's general trends have fluctuated sharply between different Middle Eastern communities [30]. However, it is important to note that CM is religiously allowed in Islam but not encouraged [31,32].

As a Muslim-dominant country in South Asia, CM is practiced in Bangladesh [24,25,33]. However, the prevalence, causal factors, and genetic and reproductive consequences had not been studied thoroughly [24,25]. The necessity of population-based surveys to compare the frequency of disorders in children of CM-couples with that of non-CM parents was described previously [34]. Some small-scale studies, particularly in the Southeastern part of the country, revealed that the prevalence of consanguinity in these areas was as high as 17.6% [10,35]. In the present study, we adopted a multiple indicator cluster survey strategy to determine that the prevalence of CM in Bangladesh is 6.638% (**Tables 2** and **S2**). The highest prevalence of this custom was observed in the Sylhet division (**S2 Table**), which is perhaps because the inhabitants of Sylhet have a traditional tendency of migrating abroad, especially to London, and marital decisions are often influenced by the chance of migration [36]. In this context, many people in Sylhet tend to marry within close relatives.

On the other hand, CM was comparatively less frequent in the Khulna area (**S2 Table**), which may be due to comparatively less favorable climatic factors for agriculture. It has been reported that Khulna and Rajshahi divisions (also Rangpur) of Bangladesh are somewhat less popular for agriculture [37]. It has been long thought that consanguinity is most common in the context of agricultural societies. Since agriculture is comparatively less widespread in the Central (Dhaka and Mymensingh) and Northern (Sylhet and Chattogram) region of Bangladesh, it can be a reason for the lower prevalence in these regions. The difference between the prevalence of CM in urban and rural areas in Khulna is insignificant (**S2 Table**), further strengthening this hypothesis. Nonetheless, we observed a significant difference in the prevalence values obtained from Khulna, Rajshahi, and Rangpur (p < 0.05). The prevalence of CM in Bangladesh is relatively lower than in most Middle Eastern countries [10, 29]. However, this study found CM a widespread practice as one in every 15 marital unions was consanguineously related.

Several studies have suggested that first-cousin marriage is the most favored type of CM among the Muslims [10,38–41]. However, the prevalence and types of CM can differ among geographic origins, even within a specific country [10,38–40,42]. Different religious, ethnic, and local or tribal traditions play significant roles behind the preference of particular types of CM [38,41,43]. In the present study, the prevalence of first-cousin marriages was 4.394%, which alone accounts for almost two-thirds of all CM cases investigated (**Table 2**). Although there was no significant difference in the preferences for any specific subtypes of first-cousin marriages; however, the aggregate of parallel and cross matrilateral marriages (52.23%) was more frequent than patrilateral unions (**Table 2**) (*p* < 0.05). In any male dominant Muslim culture, most marriage decisions come from the patrilateral side, and a robust patrivirilocal pattern of marriage is usually present [7,44]. Consequently, it is generally perceived that the dominance of patriarchal traditions and customs would drive marriage patterns in these communities, and patrilateral CM would be more prevalent than matrilateral unions. The marriage culture in Bangladesh is not a difference, an influential patrivirilocal ascendancy is present in this country, and technically, most of the CM cases were supposed to be of patrilateral types. Hence, the lack of preference for patrilateral marriages in this study was surprising (**Table 2**).

In the past few decades, Bangladesh has experienced a noticeable increase in the level of urbanization and nuclear families. Our study reflects that CM is common in both urban and rural areas of the country. However, the prevalence in rural areas is higher than in urban areas. Although this practice is mostly preferred in extended families, it is not uncommon in nuclear families (**Fig 2**). A large portion of the CM families is dependent on agriculture for their livelihood; while it is also widespread in other occupations (**Fig 2**). These outcomes indicate that the origin of this customary practice is deep-rooted in the cultural values of Bangladeshi society.

The present study suggests a strong religious bias of CM as 3693 of 3694 CM cases we investigated were followers of Islam. Only one case was observed in the Sylhet division who were believers of Hinduism. This outcome supports the conclusions of previous studies that CM is a hallmark of Muslim marital patterns [7,10,29,45]. Also, we did not find any instances of uncle-niece or aunt-nephew marriage in our studied population.

Several reports suggest that CM is more likely to be stable than non-CM, and the proportion of polygynous marriage is lower [44,45]. However, in the present study, we found that the proportion of polygynous marriage and more than once marriage is relatively higher in the CM groups (**Fig 2**). Predominantly, both polygynous and more than once marriage cases were reported from rural areas, and from within the families who depend more on agriculture for their livelihood earning. However, it is difficult to conclude if CM is less stable than its non-CM counterparts. As polygynous and multiple-marriage is a common feature of agriculture-dependent societies, this could be a reason for the higher values obtained for CM [46]. More CM families in this study were involved with agriculture-related occupations than non-CM families (**Fig 2**). We intended to evaluate the trend of divorce among CM and non-CM couples; however, many people in rural areas, especially women, tend to hide about their divorce incidents [47,48]. As a result, we could not precisely compare the divorce rate between CM and non-CM couples. The inclusion of the assessment of the divorce trends in the studied population would help evaluate CM's stability in Bangladesh.

Many studies report the negative association between consanguinity and women education in Muslim countries [49,50]. In Bangladesh, both men and women's education showed effects on consanguinity. A post-secondary or higher level of education in both men and women was significantly lower in the CM groups (**Fig 2**). Besides, a significantly higher proportion of CM females received no schooling. Overall, the rates of CM decreases with increasing levels of education. This result goes in line with previous outcomes from most Arabian and Muslim countries, and India; however, it differs from the outcomes from Oman [10,33,44,45,51]. However, a recent study on the educated-married individuals in Riyadh showed no association between participants' education level and CM [52].

We observed a significant association between socioeconomic status, e.g., education, employment, and the incidence of CM (*p* < 0.05) (**Fig 2**). Education and employment make women better able to make their own decisions [45]. On the other hand, it makes men more self-conscious and prospective. As a result, education and employment make people less interested in CM. Similar outcomes were reported in most of the Arab countries and India; however, it does not align with outcomes documented from contemporary Iran [45,51,53–55]. Interestingly, we observed no or little association between the background wealth index of the families and CM (**Fig 2**), which was previously reported by many studies [45,50,56,57]. Perhaps the intricate nature wealth index as a variable lead us to the lack of statistically insignificant association between consanguinity and wealth index.

CM plays a significant role in the reproductive behavior of people living in societies where endogamy is a common phenomenon [6,14,58,59]. In our study, we observed a cogent impact of CM on gross fertility as fertility was higher among consanguineous couples compared to their non-CM counterparts (**Fig 3**). The elevation in the gross fertility rate was predominantly owing to the birth of male offspring as compared to females, which aligns with the outcomes observed in Northern India [59]. Ober et al. reported a lower fertility rate among the CM women [60]; however, studies on Spanish, Brazilian, Icelandic, and Indian populations suggested a higher rate [59,61–63]. Our study supports the perception that CM could be a source of underlying genetic factors behind the sex-based selection of fertility [14]. It is rather difficult to correlate; nonetheless, the increased fertility in CM families could be a consequence of the motif to recompense the child-loss as both infant mortality and morbidity are higher compared to non-CM families [59,60,64]. Besides, it is unclear if the increased fertility is due to early marriage and therefore extended reproductive lives. Furthermore, the potentially improved maternal-fetal compatibility due to more shared gene loci from a CM could cause elevated fertility rates [11,65].

Many population-based reports back the assumption that CM and infant mortality are closely correlated [11,55,59,66–69]. In our study, the U5 mortality rate was higher in the CM families than in the non-CM families (**Fig 4**). Across the different sociodemographic groups, we observed elevated rates of U5 mortality of male children in the CM families. Interestingly, increases in the U5 mortality correlates with the increasing degrees of inbreeding (**Table 3 and Fig 7**). A small scale study in the Rajshahi region of Bangladesh reported similar conclusions as they found that pregnancy loss and infant mortality were highly associated with consanguinity [24]. Comparable outcomes were observed in the Jammu region of the Indian union territory. The underlying molecular basis of sex-specific infant mortality is not established yet. This study puts forward to the necessity of further investigations in explaining this association.

The sociodemographic background of a community directly influences the SSR. Sex-biased infant mortality, maternal working status, physiological indices (e.g., hormonal, nutritional), and geographical disparities can potentially influence the SSR [70–72]. Besides, heritable factors can impact SSR [73]. The SSR is considered an indicator of a significant health burden [72]. Irrespective of sociodemographic factors, our study provides evidence of a cogent repercussion on SSR due to CM (**Fig 5**). Besides, this study reports a credible effect of CM on selection intensity as the Crow's SI values were higher in CM across the sociodemographic backgrounds of the families (**Fig 6**). However, the obtained values of *I* were relatively small. What it translates into is that if there is any chance that an offspring can potentially inherit a genetic defect, it is less likely that natural selection would eliminate it. Previous studies from different parts of Bangladesh's neighboring country, India, also reported lower selection intensities among CM families [59,74]. The association between selection intensities and different degrees of CM has not been studied extensively. The outcomes of this study indicate that this association needs to be studied at a larger scale.

The LOC is a measure of the extent that refers to the relative fitness of the CM population that changes due to natural selection acting on genotypic differences from the non-CM population [75]. In our study, for each CM category, higher values of LEG were detected for autosomal inheritance as compared to X-linked inheritances, which remained the same if LEG is normalized by the inbreeding coefficient (**Table 4**). Also, it depicts that homozygosity of autosomal recessive loci could lead to deleterious consequences resulting in a high LOC. The relationship between mutations in specific genes and embryonic lethality in CM families has been proposed previously by Saudi Arabian scientists [76]. The findings of this study align with the proposed model of the link between defective genes and embryonic lethality. On a different note, double first cousins showed a lower value of LOC (**Table 4**), which might be because it is an infrequent type of CM in our studied population.

Henceforth, elevated levels of U5 mortality in the CM group demonstrate a longitudinal effect of SI and LOC. We found that the elevated gross fertility rate, SSR, and SI were directly correlated with U5 mortality and miscarriage/abortion rates in CM groups (**Figs 3, 5 and 6** and **Table 3**). It reflects the persuasive effect of natural selection to compensate for reproductive loss due to increased infant mortality and pregnancy loss. Our study depicted that the sociodemographic background had a nominal effect on reproductive behavior, while CM held a strong impact. Across sociodemographic backgrounds, CM helps to expose the disease-susceptible loci to the expression of lethal genes [14,59,69,73]. Consequently, CM has a strong influence on the reproductive behavior of men.

Consanguineous children have a relatively higher risk of having a genetic complication than those with unrelated parents [4]. Therefore, the occurrence of disease conditions with a genetic root should be higher in children from consanguineous families [77]. Previous reports have shown that some specific heritable diseases were highly perturbed in children with parents having a second cousin or closer relationship [78,79]. In countries like Bangladesh, non-communicable diseases, including genetic and multifactorial diseases, are becoming a significant cause of fatality, and it is not surprising to blame CM as one of the causes of conditions with a genetic basis. In this situation, we attempted to evaluate CM's role as a risk factor for the occurrence of genetic and multifactorial diseases.

The prevalence of hereditary disorders in the children showed a statistically significant difference between the CM and non-CM groups (**Table 5**). Notably, there was a significant difference in the prevalence between the offspring of CM versus non-CM families for all cases: bronchial asthma, disability, epilepsy, hearing defect, cardiovascular diseases, thyroidism, tumor, and vision-related problem. Surprisingly, diabetes, which is often reported to be highly perturbed in CM families, was not associated with CM [80,81]. Many previous studies from different parts of the world have shown the association between consanguinity and the diseases mentioned above [3,11,14,66,77,82–85]. Although pedigree analysis identified these diseases have a genetic root, they are generally considered as multifactorial diseases.

One of the strengths of this study is that we only dealt with the diseases having a genetic association, as per pedigree analysis revealed. Previous reports on the association of multifactorial diseases with CM families depended on the occurrence of different diseases, which might have caused inconsistency of the data. In this regard, we believe that the report this study produced is much robust than previous studies. However, finding specific genetic roots for multifactorial diseases has always been tricky and challenging [86–88]. Along with the universal diagnostic challenges, the existing infrastructural lack in Bangladesh prevents us from evaluating the genetic root of these diseases thoroughly using molecular techniques.

The importance of considering a parentally imprinted disorder was emphasized previously as children with parental consanguinity may not necessarily be affected by the autosomal recessive disorder [89]. As we mostly depended on the pedigree analysis to identify the presence of autosomal recessive disorders in this study, it is possible that we found a consanguineous couple not showing any clinical conditions, but their children presented an imprinted condition, which is recurrently reported to be an autosomal recessive disorder but not imprinted disorder, and we identified it as an autosomal recessive disorder. That is, imprinting disorder's presence may impede the analysis we did on the incidence of genetic diseases. Indeed, this study would be benefited from the inclusion of a molecular diagnostic evaluation. Also, many of the offspring we enrolled, both in the CM and non-CM group, was still in their early ages and hence the numbers for the late onset diseases may have been impacted. Nevertheless, the exceptionally high frequency of these disorders in the CM group compared to the non-CM group directs to the conclusion that there was a strong association between CM and the occurrence of these disorders.

Among the single-gene diseases, we observed a perturbation of sickle cell anemia and thalassemias in the CM group as compared to the non-CM group (**Table 5**). Furthermore, our findings are consistent with the conception that the risk of expression of an autosomal recessive disorder in the children of CM is inversely related to the frequency of carriers in the non-CM population [73]. This study indicates that there is a significant difference in the frequency of CM among the parents of individuals affected with single-gene diseases and non-CM population. A new study showed that the prevalence of CM was almost four times higher among the parents of children with ß-thalassemia [90]. A recent report from a nationwide carrier detection program revealed that the chance of being a carrier of ß-thalassemia is almost double in the children of CM couples [91].

Several reports have so far reviewed the role of CM in congenital anomalies [11,77,92]. In the present study, we observed a higher frequency of CM in the parents of children encountering congenital anomalies (**Table 6**). Notwithstanding the apparent discrepancies in the pattern and proportions of anomalies observed in this study, our outcome goes in parallel with other reports on the question. In a few reports from middle eastern countries, congenital anomalies were considerably more prevalent in the children having CM parents [77,92–95].

Congenital heart disease and physical anomalies were the most common types of congenital anomalies with almost 4-times higher frequency than their non-CM counterparts (**Table 6**). It supports the findings of two independent studies reported from Saudi Arabia, where CM was identified as a risk factor for congenital anomalies [77,96,97]. Interestingly, a similar pattern of effect on the effect of CM on the incidence of congenital anomalies was reported from Lebanon, and in the small study reported from the Rajshahi region of Bangladesh [24,98].

One important question that may arise when interpreting these data is the difference in general public health conditions between CM and non-CM families. We evaluated several public health indices among the CM and non-CM groups; however, no significant difference was observed (**Fig 8**). This finding strengthens our conclusions mentioned above- the deleterious effects observed in the CM were not due to the difference in public health conditions between the groups but consequential for CM autonomously.

The attitude of the society towards consanguineous marriages and the awareness of CM's health consequences remain largely ignored. It is reported that CM's practice is more influenced by the community's attitude rather than socioeconomic and religious issues [52]. In our studied population, most participants had poor knowledge of CM's possible hereditary burden (**Fig 9**). Only one in every 14 respondents had previous knowledge of the potential complications associated with CM. Almost half of the participants had no idea about the possibility that CM could be a risk factor for having diseased children. Shockingly, more than one-fourth (26%) of the respondents were still indifferent and having a positive attitude to CM when they came to know about the health burden of CM. Most of the people who showed a positive attitude to CM were from the older age group, males, those who are married to their relatives, and people who have a family history of CM. It was previously reported that the prevalence of CM is higher in people having a positive attitude for CM [52,99]. Our study suggests that continuous health awareness and counseling programs are needed to change a large proportion of people's attitudes towards CM.

Besides, our study revealed that CM plays an important role in the mode of marriage decision. Nearly 75% of the marriage decisions leading to a CM came from within the family and resulted in a family-decided arranged marriage. In contrast, a substantially reduced proportion of family-decided arranged marriage was observed in the non-CM families. Regardless of socioeconomic and educational backgrounds, it was apparent that more CM was due to the family choice rather than the individuals getting married. Henceforth, CM's primary reason was family choice in the context of the cultural, traditional, and religious views of the community.

Overall, this study, together with the previous studies, suggests that CM has a strong influence on reproductive behavior in the Bangladeshi population. Also, CM is associated with the occurrence of hereditary and multifactorial diseases and congenital anomalies in Bangladesh. However, the pattern and its extent of effects on the disease are, perhaps, not uniform and require further in-depth studies. This study essence the necessity of mass health education and genetic counseling programs in the country. The outcomes presented in this report should work as a baseline platform for further studies in understanding the role of CM in reproductive behavior and hereditary, multifactorial, and congenital diseases.

## Supporting information

S1 Appendix Questionnaire form for acquiesced medical data collection from consanguineous families (Bengali version). (DOCX)

S2 Appendix Methods: Definitions, measures, and procedures. (DOCX)

S1 File STROBE statement. (DOCX)

S1 Table Districts included in different zones. (DOCX)

S2 Table Prevalence of CM in Bangladesh. (DOCX)
