# Supplementary material for: Genetic and reproductive consequences of consanguineous marriage in Bangladesh
Source: PLoS One. 2020 Nov 30;15(11):e0241610. doi: 10.1371/journal.pone.0241610 (PMC7703949; doi:10.1371/journal.pone.0241610)

**S1 Appendix**

Questionnaire form for acquiesced medical data collection from consanguineous families (Bengali version).


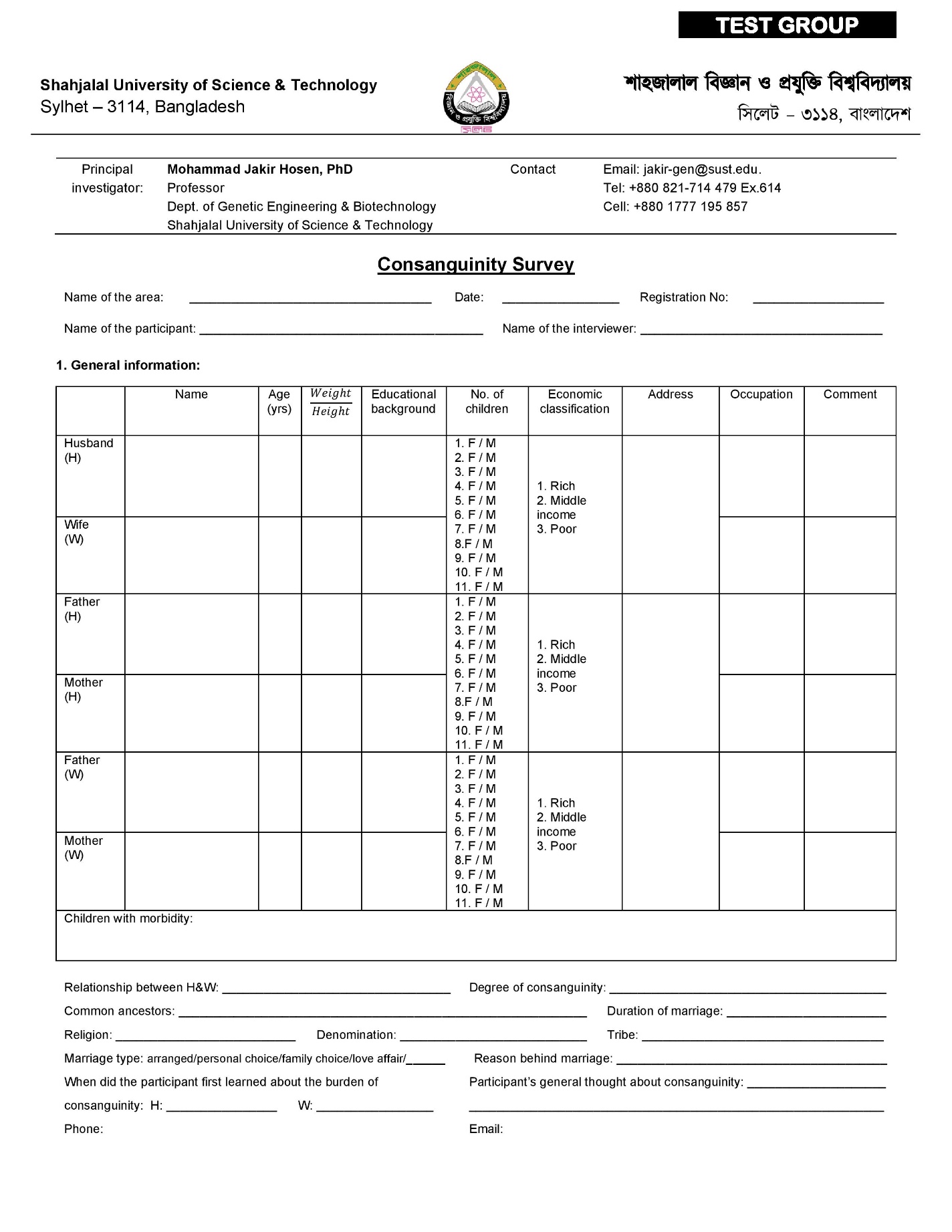


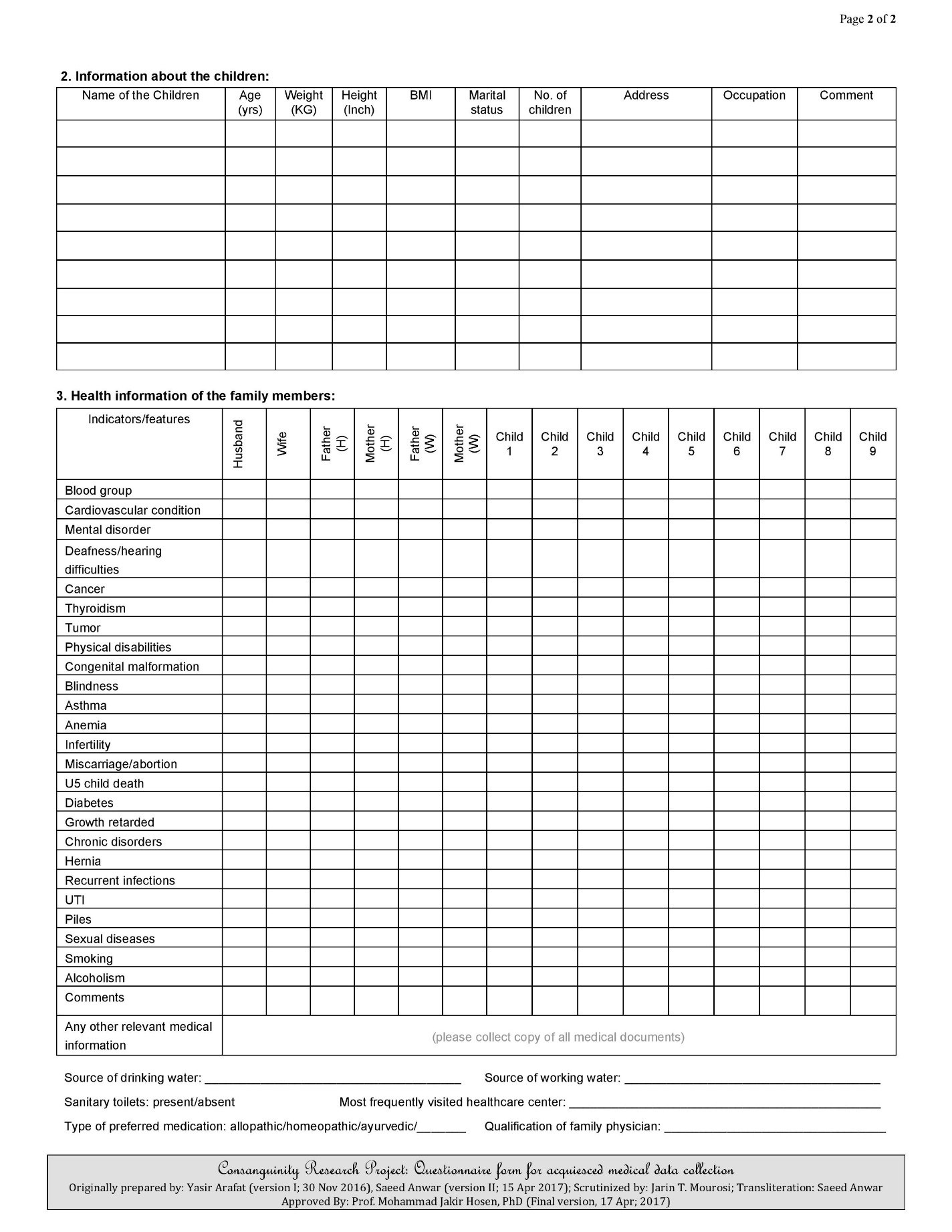

Supplement: S1 Appendix — (DOCX) [file pone.0241610.s001.docx]
