# Supplementary material for: Genetic and reproductive consequences of consanguineous marriage in Bangladesh
Source: PLoS One. 2020 Nov 30;15(11):e0241610. doi: 10.1371/journal.pone.0241610 (PMC7703949; doi:10.1371/journal.pone.0241610)
Supplement: S2 Appendix — (DOCX) [file pone.0241610.s002.docx]

**S2 Appendix**

**Questionnaire preparation and validation**

The newly prepared questionnaire was evaluated in a preliminary study. Initially, we consulted with the existing literature and prepared a draft questionnaire. Later, we asked a group of Bangladeshi health professionals and geneticists to independently assess the degree to which the questionnaire is relevant and can correctly measure the effects of consanguineous marriage (CM) in the Bangladeshi population. We then made necessary adjustments to the questionnaire to address their comments. Two independent interviewers then conducted the revised questionnaire in two locales (Jangail and Ghopal) of Sylhet. A total of 109 families (58 CM and 51 non-CM) were interviewed twice 3-months apart. The collected data were used to assess the internal consistency and test-retest reliability using Cronbach's α and intra-class correlation analysis. Both assessments indicated satisfactory levels of reliability of the questionnaire (Cronbach's α = 0.76, intra-class correlation coefficient = 0.96). Note that no data from these 109 families were included in the final analyses.

**Study area selection**

We used a stratified multi-stage cluster sampling design to sample locales in the 17 zones, where each zone was a stratum. In each of the 17 strata, a cluster was a village or a mahalla. Information on the villages and mahallas, in each district of every stratum, was collected from the Bangladesh national portal (https://www.bangladesh.gov.bd). Six clusters were systematically selected without replacement in each stratum, with at least one locale from each district. However, because we could not make any communication in 3 districts, we had to make replacements for the regions where these districts fall.

**Definitions, measures, and procedures**

*Consanguineous couples:* The married couples who reported being married to their cousin (inbreeding coefficient *≥* 0.015625) were defined as consanguineous couples.

Non-consanguineous couples: The married couples who reported not to have any close blood relations to their spouse (inbreeding coefficient ≤ 0.015625) were defined as non-consanguineous couples.

Unrelated couples: The unrelated couples were defined as any non-consanguineous couples who reported not to have any blood relations to their spouse (inbreeding coefficient < 0.0078125). Unrelated couples were enrolled in this study as controls.

Consanguineous families: A consanguineous family includes a consanguineous couple and their children.

*Non-consanguineous families:* A non-consanguineous family includes a non-consanguineous couple and their children.

*Fertility:* Fertility is the number of live births given by a mother. We determined fertility for children of both male and female sexes individually and cumulatively as well. For mothers who have completed their reproductive life, the average number of offspring born live per mother is termed the total fertility rate (TFR).

*U_5_ Mortality:* The under-five mortality rate of the offspring (U_5_ mortality) is the mortality rate among the children of the studied couples before reaching the age of five years. This estimation does not include accidental deaths (e.g., on a car road accident) of the children.

*Congenital anomalies:* Congenital anomalies were defined as a physical defect in a baby at birth, which may involve different parts of the body, including the brain, heart, lungs, liver, bones, and intestinal tract [1].

*Secondary sex ratio:* The secondary sex ratio or SSR is the ratio of males to females at birth in a population. It is the estimation of the number of boys born per 100 girls born.

*Inbreeding coefficient:* We evaluated the degree of inbreeding by calculating the probability that two alleles at a given locus, e.g., disease locus, are identical by the descent, i.e., the inbreeding coefficient (COI). Table S2 (a) represents the inbreeding coefficient in the offspring of various types of consanguineous mating [2]. Equation 1 statements the formula for calculating mean inbreeding coefficient:

$\alpha=\sum PiFi$… … … (1)

Here, Pi = the proportion of couples in each category of CM, and Fi = inbreeding coefficient of that particular category.

**Table S2 (a):** Proportion of alleles shared by couples united by CM and the inbreeding coefficient in the offspring of various types of consanguineous mating.

| **CM category** | **Proportion of shared alleles** | **Inbreeding coefficient** |
| --- | --- | --- |
| Double first cousins | 1/4 | 0.25 |
| First cousins | 1/8 | 0.125 |
| Half first cousins | 1/16 | 0.0625 |
| First cousins once removed | 1/16 | 0.0625 |
| Second cousins | 1/32 | 0.015625 |
| Second cousins once removed | 1/64 | 0.0078125 |
| Third cousins | 1/128 | 0.00390625 |

*Selection Intensity (SI):* The estimation of differential fertility and child mortality leads to the measurement of natural selection [3]. The index of selection intensity (Crow's index) is the index of opportunity for selection, which is ascribed as that the fewer the changes in the genetic makeup of a population, the lower the index of total selection intensity. Equation (2) statements the formula for calculating the Crow's index:

$\boldsymbol{I =}\frac{\boldsymbol{Pd +}\frac{\boldsymbol{Vf}}{\boldsymbol{(x̄)}^{\boldsymbol{2}}}}{\boldsymbol{1 - Pd}}$ … … … (2)

Here, I = index of total selection, P_d_ = death before reaching adulthood (18 years of age), V_f_ = variance in the number of live births due to fertility, and x̄ = average number of live births per woman until the cessation of menstruation.

*Load of consanguinity:* The Load of Consanguinity (LoC) is the rate at which fitness declines with increased inbreeding coefficient. When an offspring inherits lethal-equivalent variants in a homozygous state, the genotype potentially contributes to the documented fetal loss or childhood mortality in the cohort [4]. Equation (3) statements the formula used for determining the LoC in terms of lethal equivalents (LEs) per gamete for autosomal and sex-linked inheritance between categories:

$\frac{LE}{gametes}= \frac{\frac{Mean difference in \% mortality (non-consanguineous - consanguineous)}{100}}{F}$… … … (3)

Here, LE = Lethal equivalent, F = Inbreeding coefficient

Also, we adopted the Crow and Kimura method for calculating the inbreeding coefficient (F) for sex-linked genes [5]. Equation (4) represents the formula for calculating LoC using the Crow and Kimura method:

$LoC = \frac{S1 - S2}{F(1-S1)}$… … … (4)

Here, S1 and S2 represent the ratio of children surviving for non-CM and CM categories, respectively.

**References**:

1. Zegers-Hochschild F, Adamson GD, De Mouzon J, Ishihara O, Mansour R, Nygren K, et al. The International Committee for Monitoring Assisted Reproductive Technology (ICMART) and the World Health Organization (WHO) Revised Glossary on ART Terminology, 2009. Hum Reprod. 2009;24: 2683–2687. doi:10.1093/humrep/dep343
2. Johnston HR, Keats BJB, Sherman SL. Population Genetics. Emery and Rimoin's Principles and Practice of Medical Genetics and Genomics. Elsevier; 2019. pp. 359–373. doi:10.1016/B978-0-12-812537-3.00012-3
3. CROW JF. Some possibilities for measuring selection intensities in man. Hum Biol an Int Rec Res. 1958;30: 1–13.
4. Chakraborty R, Chakravarti A. On consanguineous marriages and the genetic load. Hum Genet. 1977;36: 47–54. doi:10.1007/BF00390435
5. Crow JE, Kimura M. An introduction to population genetics theory. Harper & Row, Publishers. New York, Evanston, and London: Harper & Row Publishers; 1970.
