# Supplementary material for: Genetic and reproductive consequences of consanguineous marriage in Bangladesh
Source: PLoS One. 2020 Nov 30;15(11):e0241610. doi: 10.1371/journal.pone.0241610 (PMC7703949; doi:10.1371/journal.pone.0241610)
Supplement: S1 File — (DOCX) [file pone.0241610.s003.docx]

**STROBE Statement**

Checklist of items that should be included in reports of ***cohort studies***

|  | Item No | Recommendation |  | |  |
| --- | --- | --- | --- | --- | --- |
| **Title and abstract** | 1 | (a) Indicate the study’s design with a commonly used term in the title or the abstract | ✓ | |  |
|  |  | (b) Provide in the abstract an informative and balanced summary of what was done and what was found | ✓ | |  |
| Introduction | | | |  | |
|  | 2 | Explain the scientific background and rationale for the investigation being reported | ✓ | |  |
|  | 3 | State specific objectives, including any prespecified hypotheses | ✓ | |  |
| Methods | | | |  | |
| Study design | 4 | Present key elements of study design early in the paper | ✓ | |  |
| Setting | 5 | Describe the setting, locations, and relevant dates, including periods of recruitment, exposure, follow-up, and data collection | ✓ | |  |
| Participants | 6 | (*a*) Give the eligibility criteria, and the sources and methods of selection of participants. Describe methods of follow-up | ✓ | |  |
|  |  | (*b*) For matched studies, give matching criteria and number of exposed and unexposed | N/A | |  |
| Variables | 7 | Clearly define all outcomes, exposures, predictors, potential confounders, and effect modifiers. Give diagnostic criteria, if applicable | ✓ | |  |
| Data sources/ measurement | 8* | For each variable of interest, give sources of data and details of methods of assessment (measurement). Describe comparability of assessment methods if there is more than one group | ✓ | |  |
| Bias | 9 | Describe any efforts to address potential sources of bias | ✓ | |  |
| Study size | 10 | Explain how the study size was arrived at | ✓ | |  |
| Quantitative variables | 11 | Explain how quantitative variables were handled in the analyses. If applicable, describe which groupings were chosen and why | ✓  + N/A | |  |
| Statistical methods | 12 | (a) Describe all statistical methods, including those used to control for confounding | ✓ | |  |
|  |  | (b) Describe any methods used to examine subgroups and interactions | ✓ | |  |
|  |  | (*c*) Explain how missing data were addressed | N/A | |  |
|  |  | (*d*) If applicable, explain how loss to follow-up was addressed | N/A | |  |
|  |  | (*e*) Describe any sensitivity analyses | ✓ | |  |
| Results | | | |  | |
| Participants | 13* | (a) Report numbers of individuals at each stage of study—eg numbers potentially eligible, examined for eligibility, confirmed eligible, included in the study, completing follow-up, and analysed | ✓ | |  |
|  |  | (b) Give reasons for non-participation at each stage | N/A | |  |
|  |  | (c) Consider use of a flow diagram |  | |  |
| Descriptive data | 14* | (a) Give characteristics of study participants (eg demographic, clinical, social) and information on exposures and potential confounders | ✓ | |  |
|  |  | (b) Indicate number of participants with missing data for each variable of interest | ✓ | |  |
|  |  | (c) Summarise follow-up time (e.g., average and total amount) | ✓ | |  |
| Outcome data | 15* | Report numbers of outcome events or summary measures over time | ✓ | |  |
| Main results | 16 | (a) Give unadjusted estimates and, if applicable, confounder-adjusted estimates and their precision (e.g., 95% confidence interval). Make clear which confounders were adjusted for and why they were included | N/A | |  |
|  |  | (b) Report category boundaries when continuous variables were categorized | ✓ | |  |
|  |  | (c) If relevant, consider translating estimates of relative risk into absolute risk for a meaningful time period | N/A | |  |
| Other analyses | 17 | Report other analyses done— e.g., analyses of subgroups and interactions, and sensitivity analyses | ✓ | |  |
| Discussion | | | |  | |
| Key results | 18 | Summarise key results with reference to study objectives | ✓ | |  |
| Limitations | 19 | Discuss limitations of the study, taking into the account sources of potential bias or imprecision. Discuss both direction and magnitude of any potential bias | ✓ | |  |
| Interpretation | 20 | Give a cautious overall interpretation of results considering objectives, limitations, multiplicity of analyses, results from similar studies, and other relevant evidence | ✓ | |  |
| Generalisability | 21 | Discuss the generalisability (external validity) of the study results | ✓ | |  |
| Other information | | | |  | |
| Funding | 22 | Give the source of funding and the role of the funders for the present study and, if applicable, for the original study on which the present article is based | ✓ | |  |
| ✓ = Done; ✗= Not done; N/A = Not applicable to this study | | | | | |

*Give information separately for cases and controls in case- control studies and, if applicable, for exposed and unexposed groups in cohort and cross- sectional studies.
