# Supplementary material for: Genetic and reproductive consequences of consanguineous marriage in Bangladesh
Source: PLoS One. 2020 Nov 30;15(11):e0241610. doi: 10.1371/journal.pone.0241610 (PMC7703949; doi:10.1371/journal.pone.0241610)
Supplement: S1 Table — (DOCX) [file pone.0241610.s004.docx]

**S1 Table:** **Districts included in different zones**. The whole country was divided in 18 zones initially; however, Chattogram hill area was excluded from our study. Hence, this study was confined within the other 17 zones. We could not conduct a survey in Kurigram, Lalmonirhat (Rangpur zone) and Meherpur (Kushtia zone).

| Sl. | **Zone** | **Included districts** | **Comments** |
| --- | --- | --- | --- |
| 1. | Dhaka | Dhaka, Narayanganj, Munshiganj, Manikganj, Gazipur |  |
| 2. | Mymensingh | Mymensingh, Netrokona, Kishoreganj |  |
| 3. | Tangail | Tangail, Sherpur, Jamalpur |  |
| 4. | Dinajpur | Dinajpur, Thakurgaon, Panchagarh, Jaypurhat |  |
| 5. | Rangpur | Rangpur, Kurigram, Lalmonirhat, Nilphamari, Gaibandha | We could not survey in: (1) Kurigram, and (2) Lalmonirhat |
| 6. | Rajshahi | Rajshahi, Natore, Naogaon, Chapainawabganj, Bogra |  |
| 7. | Jashore | Jashore, Jhenaidah, Magura, Narail |  |
| 8. | Kushtia | Kushtia, Chuadanga, Meherpur | We could not survey in: (1) Meherpur |
| 9. | Pabna | Pabna, Shirajganj, Rajbari |  |
| 10. | Khulna | Khulna, Bagerhat, Satkhira |  |
| 11. | Gopalganj | Gopalganj, Faridpur, Madaripur, Shariatpur |  |
| 12. | Barisal | Barisal, Jhalokati, Perojpur |  |
| 13. | Patuakhali | Patuakhali, Barguna, Bhola |  |
| 14. | Cumilla | Cumilla, Chandpur, Brahmanbaria |  |
| 15. | Noakhali | Noakhali, Laxmipur, Feni |  |
| 16. | Sylhet | Sylhet, Sunamganj, Habiganj, Moulvibazar |  |
| 17. | Chattogram | Chattogram, Cox’s Bazar |  |
| 18. | Chattogram hilly area | Rangamati, Bandarban, Khagrachari | We did not survey in this zone |
