# Supplementary material for: Genetic and reproductive consequences of consanguineous marriage in Bangladesh
Source: PLoS One. 2020 Nov 30;15(11):e0241610. doi: 10.1371/journal.pone.0241610 (PMC7703949; doi:10.1371/journal.pone.0241610)
Supplement: S2 Table — (DOCX) [file pone.0241610.s005.docx]

**S2 Table:** Prevalence of CM in Bangladesh.

| **Location** | **CM** | | |  | **Non-CM** | | **Total number**  **(n)** |
| --- | --- | --- | --- | --- | --- | --- | --- |
|  | **Number**  **(n)** | **Percentage**  **(%)** | **Mean inbreeding coefficient** |  | **Number**  **(n)** | **Percentage**  **(%)** |  |
| Sylhet |  |  |  |  |  |  |  |
| Urban | 258 | 8.758 | 0.0514 |  | 2688 | 91.242 | 2946 |
| Rural | 439 | 9.519 | 0.0499 |  | 4173 | 90.481 | 4612 |
| Total | 697 | 9.222 | 0.0505 |  | 6861 | 90.778 | 7558 |
| Rangpur |  |  |  |  |  |  |  |
| Urban | 193 | 5.942 | 0.04885 |  | 3055 | 94.058 | 3248 |
| Rural | 226 | 6.892 | 0.0483 |  | 3053 | 93.108 | 3279 |
| Total | 419 | 6.419 | 0.0485 |  | 6108 | 93.581 | 6527 |
| Rajshahi |  |  |  |  |  |  |  |
| Urban | 190 | 5.766 | 0.0486 |  | 3105 | 94.234 | 3295 |
| Rural | 218 | 6.55 | 0.0497 |  | 3110 | 93.45 | 3328 |
| Total | 408 | 6.16 | 0.0492 |  | 6215 | 93.84 | 6623 |
| Mymensingh |  |  |  |  |  |  |  |
| Urban | 264 | 7.639 | 0.0492 |  | 3192 | 92.361 | 3456 |
| Rural | 310 | 8.852 | 0.0466 |  | 3192 | 91.148 | 3502 |
| Total | 574 | 8.249 | 0.0478 |  | 6384 | 91.751 | 6958 |
| Khulna |  |  |  |  |  |  |  |
| Urban | 116 | 3.306 | 0.0494 |  | 3393 | 96.694 | 3509 |
| Rural | 112 | 3.146 | 0.048 |  | 3448 | 96.854 | 3560 |
| Total | 228 | 3.333 | 0.0487 |  | 6841 | 96.667 | 7069 |
| Dhaka |  |  |  |  |  |  |  |
| Urban | 232 | 5.809 | 0.0478 |  | 3764 | 94.194 | 3996 |
| Rural | 211 | 5.84 | 0.0486 |  | 3402 | 94.16 | 3613 |
| Total | 443 | 5.822 | 0.0482 |  | 7166 | 94.178 | 7609 |
| Chattogram |  |  |  |  |  |  |  |
| Urban | 210 | 6.54 | 0.0508 |  | 3001 | 93.46 | 3211 |
| Rural | 211 | 6.52 | 0.0501 |  | 3025 | 93.48 | 3236 |
| Total | 421 | 6.53 | 0.0505 |  | 6026 | 93.47 | 6447 |
| Barishal |  |  |  |  |  |  |  |
| Urban | 226 | 6.744 | 0.0495 |  | 3125 | 93.256 | 3351 |
| Rural | 278 | 7.934 | 0.0509 |  | 3226 | 92.066 | 3504 |
| Total | 504 | 7.352 | 0.0503 |  | 6351 | 92.648 | 6855 |
| Bangladesh |  |  |  |  |  |  |  |
| Urban | 1669 | 6.183 | 0.0494 |  | 25323 | 93.817 | 26992 |
| Rural | 2025 | 7.067 | 0.0489 |  | 26629 | 92.932 | 28652 |
| Total | 3694 | 6.638 | 0.0492 |  | 51952 | 93.362 | 55646 |
